# Copper Oxide Nanoparticles Induced Growth and Physio-Biochemical Changes in Maize (*Zea mays* L.) in Saline Soil

**DOI:** 10.3390/plants13081080

**Published:** 2024-04-11

**Authors:** Hina Shafiq, Muhammad Yousaf Shani, Muhammad Yasin Ashraf, Francesco De Mastro, Claudio Cocozza, Shahid Abbas, Naila Ali, Zaib-un-Nisa, Aqsa Tahir, Muhammad Iqbal, Zafran Khan, Nimra Gul, Gennaro Brunetti

**Affiliations:** 1 Institute of Molecular Biology and Biotechnology, The University of Lahore, Lahore 54590, Pakistan; hinashafiq25@gmail.com (H.S.); nailaali@imbb.uol.pk (N.A.); zaibnisa@imbb.uol.pk (Z.-u.-N.); 2 Pakistan Institute of Engineering and Applied Sciences (PIEAS), Nuclear Institute for Agriculture and Biology College (NIAB-C), Islamabad 45650, Pakistan; mmyousafshani@gmail.com; 3 Plant Breeding and Genetics Division, Nuclear Institute for Agriculture and Biology (NIAB), Faisalabad 38000, Pakistan; 4 Department of Soil, Plant, and Food Sciences, University of Bari “Aldo Moro”, 70126 Bari, Italy; claudio.cocozza@uniba.it (C.C.); gennaro.brunetti@uniba.it (G.B.); 5 Institute of Soil and Environmental Sciences, University of Agriculture, Faisalabad 38000, Pakistan; shahidsail321@gmail.com (S.A.); iqbal@uaf.edu.pk (M.I.); 6 Department of Agricultural Engineering, National University of Sciences and Technology, Islamabad 45650, Pakistan; aqsatahir290@gmail.com; 7 Department of Plant Breeding and Genetics, University of Agriculture Faisalabad, Faisalabad 38000, Pakistan; zafrankhan@gmail.com (Z.K.); nimra122@gmail.com (N.G.)

**Keywords:** salinity stress, nanoparticles, copper oxide, maize crop, foliar application

## Abstract

Research on nanoparticles (NPs) is gaining great attention in modulating abiotic stress tolerance and improving crop productivity. Therefore, this investigation was carried out to evaluate the effects of copper oxide nanoparticles (CuO-NPs) on growth and biochemical characteristics in two maize hybrids (YH-5427 and FH-1046) grown under normal conditions or subjected to saline stress. A pot-culture experiment was carried out in the Botanical Research Area of “the University of Lahore”, Lahore, Pakistan, in a completely randomized design. At two phenological stages, both maize hybrids were irrigated with the same amount of distilled water or NaCl solution (EC = 5 dS m^−1^) and subjected or not to foliar treatment with a suspension of CuO-NPs. The salt stress significantly reduced the photosynthetic parameters (photosynthetic rate, transpiration, stomatal conductance), while the sodium content in the shoot and root increased. The foliar spray with CuO-NPs improved the growth and photosynthetic attributes, along with the N, P, K, Ca, and Mg content in the roots and shoots. However, the maize hybrid YH-5427 responded better than the other hybrid to the saline stress when sprayed with CuO-NPs. Overall, the findings of the current investigation demonstrated that CuO-NPs can help to reduce the adverse effects of salinity stress on maize plants by improving growth and physio-biochemical attributes.

## 1. Introduction

Globally, salinity stress is a vital restricting factor and a major problem for crop production. Salinity is the most common abiotic stress in arid and semi-arid areas of the world [1]. Across the globe, 800 million hectares of land are saline [2,3], while in Pakistan, almost 6.8 million hectares are negatively affected by salts [4]. Salinity adversely affects the morpho-physiological attributes of plants, consequently reducing their yield [5]. In irrigated areas, plant growth and productivity decreased due to osmotic stress caused by salinity [6]. Salinity stress causes alterations in water absorption and disrupts the ionic content in plants [7]. An excessive concentration of salts in the soil reduces photosynthetic activity by reducing the area of the leaf and the chlorophyll content that results in early leaf senescence [8]. Salinity stress causes a biochemical change due to hyper-ionic [9], osmotic stress [7], membrane injury [10], reduction in nutrient uptake [11,12], disturbance of plant hormone secretions [13], enzyme activities [14] and metabolic disorders [15].

Worldwide, the third most important cereal crop is maize (*Zea mays* L.) after wheat and rice. Due to its high nutritive values, it is grown throughout the world and has a higher genetic diversity among cereals [16]. Maize is a C4 crop, with a wide genetic variability that allows its development in a wide range of soils and under different climatic conditions [17]. The productivity of the maize crop is adversely affected by various abiotic stresses [18]. For the proper growth and development of maize crops, an efficient nutrient management system is in urgent need [19].

Copper (Cu) is one of the eight essential micronutrients and had a pivotal contribution in the synthesis of metalloproteins where it acts as a cofactor in the regulation of enzyme activities [20]. It has a decisive role in regulating the amalgamation of different macromolecules that have cardinal metabolic activities in plants, including photosynthesis, respiration, lignification of cell walls, and different defense systems against abiotic stresses [21]. Copper plays an imperative role in controlling plant growth and development, including chlorophyll content and seed dormancy [22]. The productivity of maize is sensitive to Cu deficiency because copper plays an important role in its growth and development [23]. It is also needed during the time spent in photosynthesis, which is fundamental for respiration and the metabolism of carbohydrates and proteins in plants [24]. The use of Cu nanoparticles (Cu-NPs) showed an impact on seed yield and quality in soybeans [25].

The impact of various levels of Cu on germination, seedling development, and physiological parameters is well documented in the literature, but contradictory [26]. Copper nanoparticles are applied effectively in numerous fields, for example, in antimicrobials and fungicides for the protection of horticultural crops [27]. Phytotoxicity of Cu-NPs is also identified as having a negative effect on metabolism in plants. Copper nanoparticles decreased sugar substance and increased lipid peroxidation, which plays a significant role in plant yield and also decreases the hindrance to root development and plant biomass of *A. thaliana* [28]. Copper nanoparticles improve the development and yield of wheat and do not limit the seed germination of barley [29].

Considering the adverse effect of salinity, the importance of maize as a food crop, and the involvement of Cu in different metabolic activities, a research project was initiated to investigate the effect of the foliar application of copper oxide nanoparticles (CuO-NPs) on morpho-physiological and biochemical parameters of maize plants under salinity stress.

## 2. Results

### 2.1. Growth Parameters

**Figure 1:**
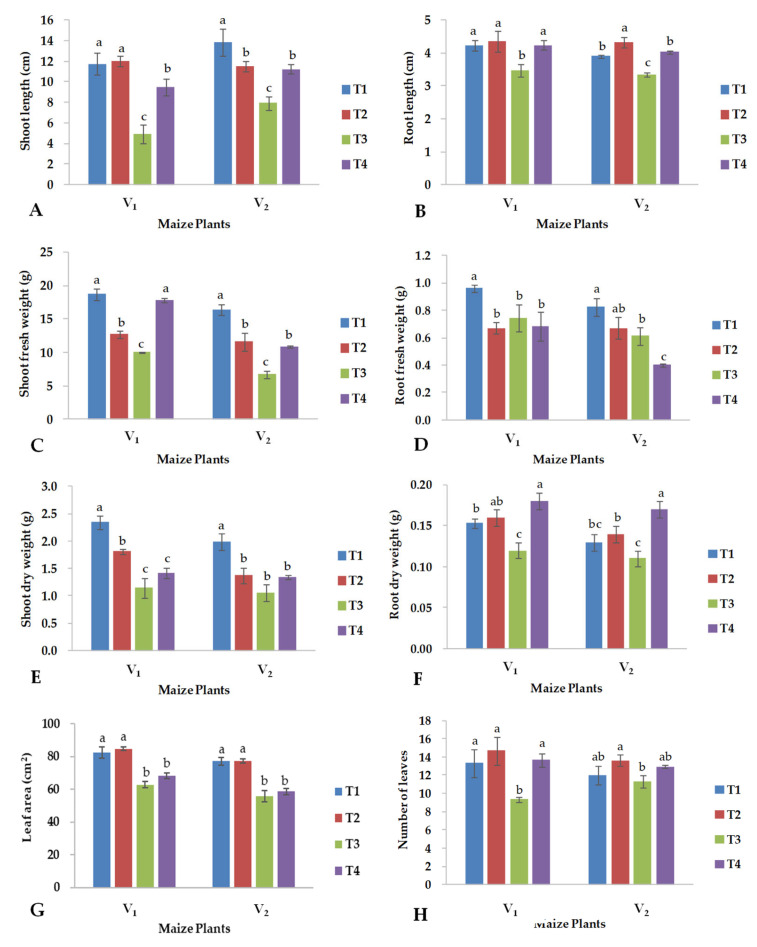
Effects of copper oxide nanoparticles and salinity stress on (**A**) shoot length, (**B**) root length, (**C**) shoot fresh weight, (**D**) root fresh weight, (**E**) shoot dry weight, (**F**) root dry weight, (**G**) leaf area index, (**H**) number of leaves in maize hybrid YH-5427 (V_1_) and FH-1046 (V_2_). T1: control; T2: foliar CuO-NPs; T3: salinity stress; T4: salinity stress + foliar CuO-NPs. Different letters demonstrate significant differences among the treatments (*p* < 0.05).

Salinity significantly influenced the growth of maize plants by reducing all morphological parameters studied. However, the foliar application of CuO-NPs, especially under saline conditions, had positive effects on almost all morphological parameters in both maize hybrids. Specifically, with the application of CuO-NPs under saline conditions, the shoot length of hybrid YH-5427 (V_1_) increased by 93%, while in hybrid FH-1046 (V_2_), it improved by 41% relative to their corresponding controls (Figure 1A). Similarly, the application of CuO-NPs produced a significant improvement in the root length of both the hybrids under saline conditions (Figure 1B): it was 22.4% for YH-5427 and 19.8% for FH-1046; while under control conditions, the foliar application of CuO-NPs also resulted in a significant increase (11.1%) of root length only for FH-1046 (Figure 1B). The application of CuO-NPs significantly increased the fresh weight of the shoot of both hybrids under saline conditions (Figure 1C). Under the same treatment, only the dry weight of the roots increased significantly with the foliar application of CuO-NPs in both maize hybrids with respect to control conditions (Figure 1F), while no significant effect was observed for the dry weight of the shoot (Figure 1E). Moreover, a significantly lower number of leaves has been recorded for both hybrids grown under saline conditions without the application of CuO-NPs (Figure 1H), while no effect of the foliar treatment was observed for the leaf area index under all conditions (Figure 1G). However, salinity significantly reduced the leaf area of both hybrids.

The three-way interaction among salinity (S), variety (V) and nanoparticles (S × V × CuO-NPs) was not significant for all the attributes mentioned above, with the exception of leaf area, as reported in Table 1.

### 2.2. Biochemical Parameters

**Figure 2:**
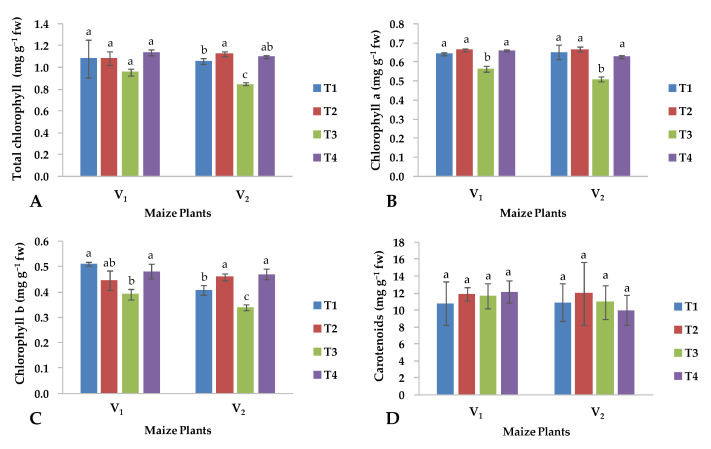
Effects of copper oxide nanoparticles and salinity stress on (**A**) total chlorophyll, (**B**) chlorophyll a, (**C**) chlorophyll b, (**D**) carotenoids in both maize hybrids (V_1_: YH-5427; V_2_: FH-1046). T1: control; T2: foliar CuO-NPs; T3: salinity stress; T4: salinity stress + foliar CuO-NPs. Different letters demonstrate significant differences among the treatments (*p* < 0.05).

In general, salinity influenced the concentration of total chlorophyll (T Chl), chlorophyll a (Chl a), and chlorophyll b (Chl b) in a similar manner, except for the carotenoids (Car) content, which was not affected by salinity in both maize hybrids (Figure 2). In particular, under saline conditions, all chlorophylls reduced their concentration, but the foliar application of CuO-NPs returned the content of all chlorophylls to the same values as those observed under control conditions (Figure 2A–C). However, three-way interaction among S × V × CuO-NPs was not significant among all traits mentioned above, except for the Chl b content (Table 1).

**Figure 3:**
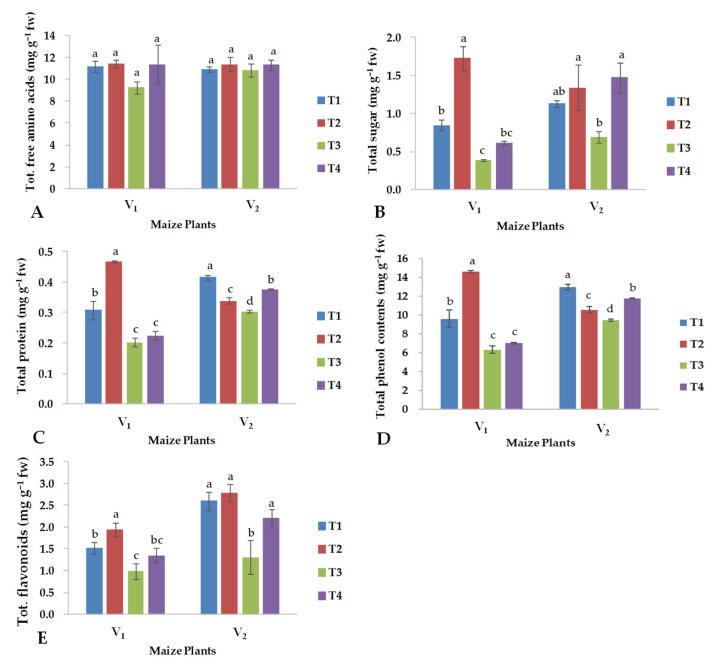
Effects of copper oxide nanoparticles and salinity stress on (**A**) total free amino acids, (**B**) total sugars, (**C**) total protein, (**D**) total phenol and (**E**) total flavonoids in both maize hybrids (V_1_: YH-5427; V_2_: FH-1046). T1: control; T2: foliar CuO-NPs; T3: salinity stress; T4: salinity stress + foliar CuO-NPs. Different letters demonstrate significant differences among the treatments (*p* < 0.05).

Total soluble sugars (TS), total protein (TP), total phenol content (TPC) and, to a lesser extent, total flavonoid (TFAL) were significantly and negatively affected by salinity stress in both varieties of maize, except for total free amino acids (TFAAs) (Figure 3). Under control conditions, the hybrid YH-5427 benefited from the foliar application of CuO-NPs for TS (Figure 3B), TP (Figure 3C), TPC (Figure 3D), and TFAL (Figure 3E), while the other maize hybrid (FH-1046) almost showed a similar trend except for TP and TPC, which were significantly reduced with the CuO-NPs spray treatment (Figure 3C,D), respectively.

Under saline conditions, the maize hybrid FH-1046 benefited more than the other maize hybrid, YH-5427, from the foliar application of CuO-NPs considering the parameters TS, TPC, TP and TFAL. The three-way interaction among S × V × CuO-NPs was significant for all biochemical parameters, with the exception of TFAL (Table 2).

### 2.3. Nutrient Content in Shoot

**Figure 4:**
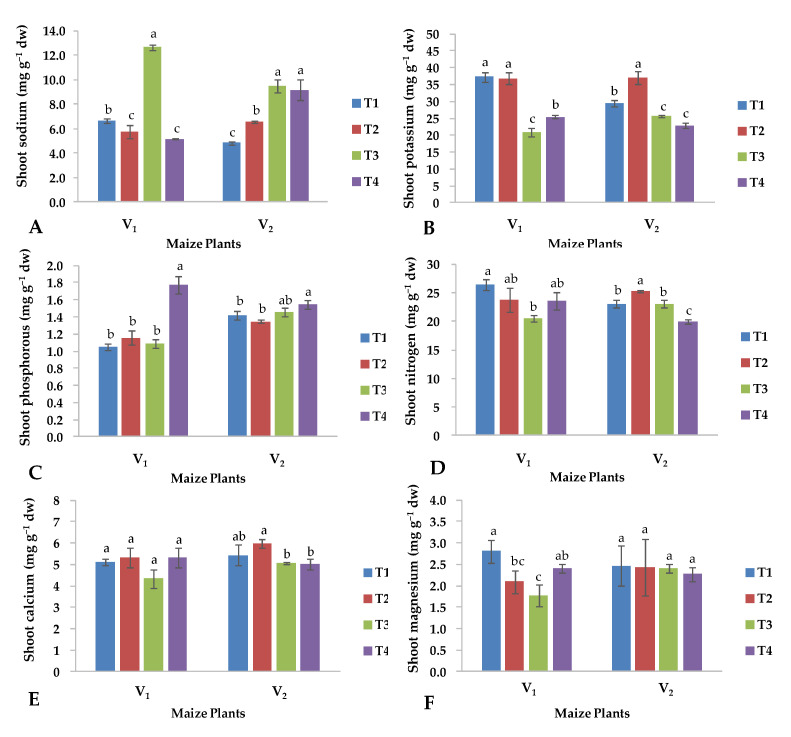
Effects of copper oxide nanoparticles and salinity stress on (**A**) total sodium, (**B**) total potassium, (**C**) total phosphorus, (**D**) total nitrogen, (**E**) total calcium, (**F**) total magnesium, in shoots of both maize hybrids (V_1_: YH-5427; V_2_: FH-1046). T1: control; T2: foliar CuO-NPs; T3: salinity stress; T4: salinity stress + foliar CuO-NPs. Different letters demonstrate significant differences among the treatments (*p* < 0.05).

The elemental composition of the FH-1046 hybrid shoot (V_2_) grown under control conditions was significantly influenced by the application of CuO-NPs, except P and Mg that did not show any significant change (Figure 4). In fact, the foliar treatment of CuO-NPs increased the content of Na, P, N, and Ca under control conditions. With regard to the other maize hybrid YH-5427 (V_1_), the application of CuO-NPs almost did not affect the content of K, P and Ca of the shoot, while a decreasing trend of Na, N and Mg content was observed.

Under saline conditions, the maize hybrid “YH-5427” showed a lower content of K, P, N, Ca and Mg in the shoot than the maize hybrid FH-1046, apart from the Na content. The application of CuO-NPs under saline conditions mainly affected the YH-5427 hybrid (V_1_), whose concentrations of K, P, N, Ca and Mg increased, while the Na content decreased noticeably. The hybrid FH-1046 did not show any significant change under the same conditions, except for N, whose concentration was reduced (Figure 4).

### 2.4. Nutrient Contents in Roots

**Figure 5:**
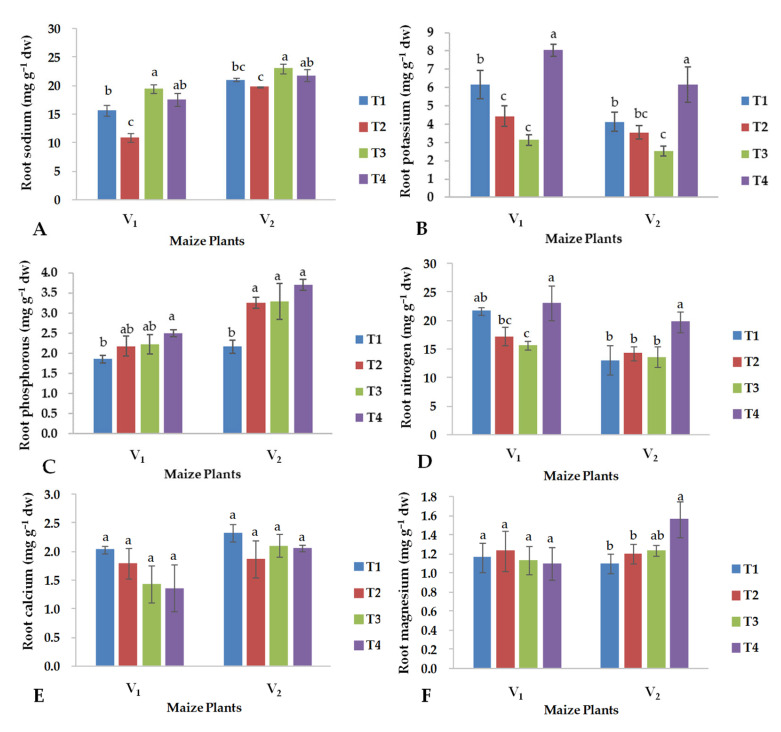
Effects of copper oxide nanoparticles and salinity stress on (**A**) total sodium, (**B**) total potassium, (**C**) total phosphorus, (**D**) total nitrogen, (**E**) total calcium, (**F**) total magnesium, contents in roots of two maize hybrids (V_1_: YH-5427; V_2_: FH-1046). T1: control; T2: foliar CuO-NPs; T3: salinity stress; T4: salinity stress + foliar CuO-NPs. Different letters indicate significant differences among the treatments (*p* < 0.05).

The maize hybrid YH-5427 (V_1_) grown in control soil suffered from the application of CuO-NPs since the contents of Na, K and N were reduced compared to the untreated plants (Figure 5). In contrast, the foliar application of CuO-NPs did not influence the Mg and P content of the same hybrid under control conditions (Figure 5C,F). The elemental composition of the FH-1046 maize root was similar between the untreated and treated control plants, except for P, which increased with the foliar application of CuO-NPs.

Under saline conditions, the K and N content of the root of both hybrids was low in untreated plants, while the foliar application of CuO-NPs increased the contents of these nutrients, and their concentrations were higher in the maize hybrid “YH-5427” than in FH-1046 (Figure 5B,D). In contrast, an opposite trend was observed for the content of Na, P, Mg and Ca, since the FH-1046 maize hybrid (V_2_) showed a higher concentration of the elements mentioned above when treated with the CuO-NPs (Figure 5A,C,E,F).

**Table 1:** Means sum of squares for growth, physio-biochemical attributes under different treatments for two maize hybrids V_1_: YH-5427 and V_2_: FH-1046.

Source	DF	SL	RL	SFW	SDW	RFW	RDW	LAI	NOL	T.Chl	Chl a	Chl b	Car	TFAA	TSS	TSP	TPC	T.FAL
Variety	1	15.360 ***	0.260	0.110	0.032	0.051	0.014	597.600 ***	35.375 **	0.007	0.002 *	0.009 ***	2.863	7.116 *	0.423 ***	0.020 ***	19.429 **	3.600 **
Salinity	1	90.090 ***	1.000	75.590 *	5.587 ***	0.111	0.002 ^ns^	4291.500 ***	15.042 *	0.035 **	0.026 ***	0.007 ***	0.238	2.537	1.330 ***	0.066 ***	64.908 ***	3.353 **
CuO-NPs	1	12.610 ***	3.154	0.389	1.540 **	0.002	0.001	289.820 **	18.375 *	0.096 ***	0.023 ***	0.017 ***	1.212	16.454 ***	1.649 ***	0.012 **	11.646 **	1.279 *
V × S	1	3.840 *	0.220	19.234	0.047	0.057	0.003	719.850 ***	5.042	0.0115	0.004 ***	0.0002	3.347	0.727	0.594 ***	0.028 **	27.695 **	0.212
V × CuONPs	1	5.704 *	0.634	51.202 *	0.070	0.496	0.005	500.230 ***	22.042 *	0.007	0.0001	0.010 ***	0.872	1.397	0.006	0.013 **	12.624 **	0.033
S × CuO-NPs	1	37.500 ***	0.350	193.546 ***	3.375 ***	0.634	0.029	2202.630 ***	9.375	0.050 **	0.012 ***	0.021 ***	2.529	0.002	0.002	0.0001	0.056	0.153
V × S × CuO-NPs	1	0.634 ^ns^	0.004 ^ns^	32.469	0.180 ^ns^	0.012 ^ns^	0.004 ^ns^	1393.090 ***	9.375 ^ns^	1.064 ^ns^	0.0003 ^ns^	0.002 *	0.768 ^ns^	5.177 *	0.555 ***	0.031 ***	30.445 ***	0.222 ^ns^
Error	14	0.706	0.789	10.372	0.172	0.252	0.012	22.230	2.696	0.005	0.00024	0.0004	4.450	0.759	0.014	0.001	1.178	0.278

* indicates significance differences among the treatments *p* > 0.001 ***, *p* < 0.01 **, (0.01< *p* ≥ 0.05) *, *p* ≥ 0.05, ^ns^ (non-significant); SL, shoot length; RL, root length; SFW, shoot fresh weight; SDW, shoot dry weight; RFW, root fresh weight; RDW, root dry weight; LAI, leaf area index; NOL, number of leaves/per plant; total chlorophyll. (T.Chl); chlorophyll a (Chl a); chlorophyll b (Chl b); carotenoids (Car); total free amino acids (TFAA); total soluble sugars (TSS); total soluble proteins (TSP), total phenolic content (TPC); total flavonoids (T.FAL).

**Table 2:** Mean sum of squares for nutrient content estimation under different treatments for two maize hybrids V_1_: YH-5427 and V_2_: FH-1046.

Source	DF	NaS	KS	PS	NS	CaS	MgS	NaR	KR	PR	NR	CaR	MgR
Variety	1	0.179	11.760 *	0.178 ***	0.031	0.540 *	0.094	182.216 ***	15.058 ***	5.006 ***	1.076 ***	1.175 **	0.082
Salinity	1	50.605 ***	780.900 ***	0.296 ***	0.510 ***	2.042 **	0.350 ^ns^	77.940 ***	2.581 *	1.929 **	0.129	0.398 *	0.042
CuO-NPs	1	24.221 ***	31.099 ***	0.240 ***	0.0003	1.402 **	0.020	31.855 **	19.820 ***	1.657 **	0.404 **	0.185	0.082
V × S	1	3.293	34.656 ***	0.067 **	0.003	0.042	0.094	15.730 *	0.128	0.297	0.148	0.353	0.167 *
V × CuO-NPs	1	44.200 ***	0.228	0.227 ***	0.006	0.282	0.004	6.816	0.519	0.320	0.072	0.011	0.060
S × CuO-NPs	1	34.297 ***	10.088 *	0.214 ***	0.00082	4.168	0.570 *	3.581	52.068 ***	0.187	1.074 ***	0.092	0.007
V × S × CuO-NPs	1	11.03 **	88.704 ***	0.067 **	0.447 ***	0.540 *	0.770 *	3.205 ^ns^	4.259 **	0.143 ^ns^	0.181 *	0.020 ^ns^	0.042 ^ns^
Error	14	0.9974	1.618	0.004	0.011	0.123	0.119	1.951	0.3237	0.137	0.037	0.086	0.024

* indicate significance differences among the treatments *p* > 0.001 ***, *p* < 0.01 **, (0.01< *p* ≥ 0.05) *, *p* ≥ 0.05, ^ns^ (non-significant); NaS, sodium content in shoot; KS, potassium content in shoot; PS, phosphorus content in shoot; NS, nitrogen content in shoot; CaS, calcium content in shoot; MgS, magnesium content in shoot; NaR, sodium content in root; KR, potassium content in root; PR, phosphorus content in root; NR, nitrogen content in root; CaR, calcium content in root; MgR, magnesium content in root.

### 2.5. Principal Component Analysis

**Figure 6:**
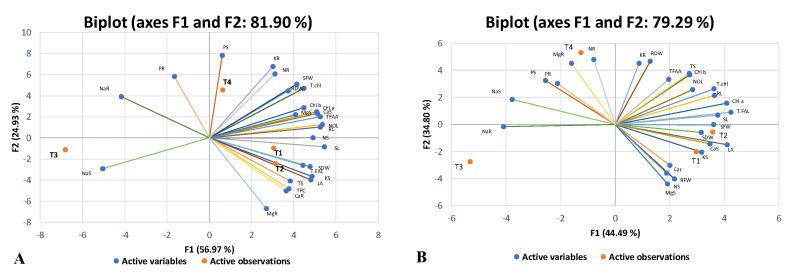
Biplots or scores value analysis of both selected maize hybrids developed via PCA analysis for two maize hybrids (**A**) V_1_: YH-5427 and (**B**) V_2_: FH-1046 for all morpho-physiological and biochemical parameters.

**Figure 7:**
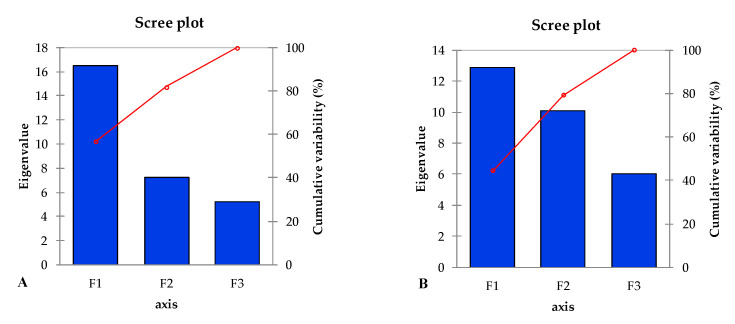
Bar charts unveiling eigenvalue and percentage of variation in commutative variability contribution by all principal components (PCs) in two different maize hybrids (**A**) V_1_: YH-5427 and (**B**) V_2_: FH-1046.

Principal component analysis (PCA) is a multivariate statistical analysis normally used to interpret large datasets. In the current investigation, loading plots of PCA were developed for both selected maize hybrids separately to assess the alterations in morpho-physiological and biochemical attributes under saline conditions and CuO-NPs application, alone or in combination, compared to the control (Figure 6A,B). The length of the vector from the centroid region of the biplot to the peripheral area of the constructed biplot showed the degree of variation among the traits studied. Between both maize hybrids, the PCA developed for YH-5427 (V_1_) revealed that the results could be explained by assessing the contribution of the first two main components, i.e., PC1 (56.97%) and PC2 (24.93%). The eigenvalues of the first three principal components (PCs) were greater than 1, but the cumulative variability of the first two PCs was (81.9%), and therefore, a biplot was constructed between the first two principal components (Figure 7A,B). The biplot analysis was performed for hybrid YH-5427 (V_1_) using XL-STAT software (version 2019) which depicted that CuO-NPs treatment (T_2_) was effective in revealing coherent synergistic variations among several attributes such as total phenolic contents (TPC), total sugars (TS), root carotenoids content (CaR) and root magnesium content (MgR) as their vector lengths were in the same direction, while these changes were antagonistic with variations in root sodium (NaR) and phosphorus (PR) contents as the vector length was laying in the opposite direction present in the first quadrant of the constructed biplot. The maize plants grown under salinity stress (T_3_) revealed noticeable alterations in sodium concentration (NaS) in the shoot of the hybrid YH-5427 and showed a negative interaction with the concentration of SFW, RDW and Chl.b. Furthermore, treatment with CuO-NPs+Salinity stress (T_4_) revealed alterations in the concentrations of several key nutrients such as shoot phosphorus (PS), root potassium (KR) and root nitrogen content (NR). However, maize plants grown under control conditions (T_1_) showed synergistic variation patterns of several imperative attributes such as dry weight of the shoot (SDW), length of the shoot (SL) and the nitrogen content of the shoot (NS) (Figure 6A).

**Figure 8:**
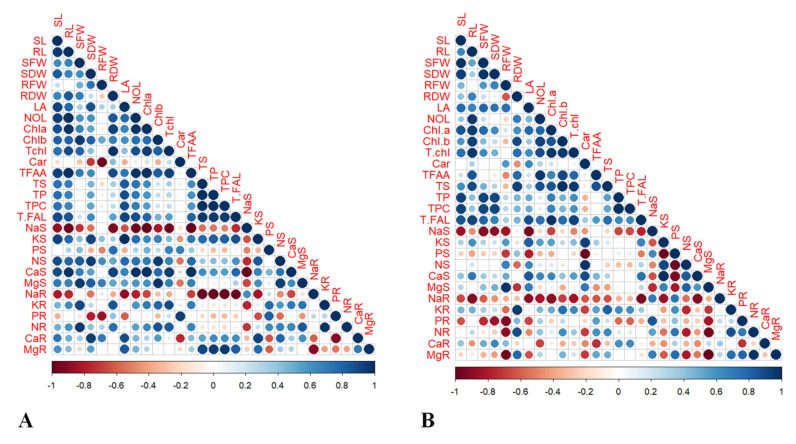
Pearson’s correlation analyses of both selected maize hybrids (**A**) YH-5427 and (**B**) FH-1046 for all studied growth, physiological and biochemical attributes.

Similarly, to better comprehend the variation in the traits studied under different treatments, another biplot was developed for the second hybrid maize FH-1046 (V_2_), which showed that the cumulative variability between the first two PCs was (79.29%) (Figure 8B). Maize plants grown under control conditions (T_1_) showed a more positive interaction for shoot potassium (KS), calcium (CaS), root fresh weight (RFW) and magnesium content in the shoot (MgS), while CuO-NPs foliar implication (T_2_) was effective in showing concomitant variations in SDW, SFW, SL and T.FAL, while these were negatively linked with alteration of the Na content of the shoot and root (Figure 6B). However, salinity stress treatment (T_3_) enhanced the root Na concentration, and these were negatively associated with the alterations in SDW, CaS and KS content. However, CuO-NPs+salinity stress (T_4_) demonstrated variations in root magnesium (MgR), root nitrogen (NR) and phosphorus contents in both root and shoot (Figure 6B).

### 2.6. Pearson’s Correlation and Heatmap Analyses

Moreover, to strengthen our research findings, heatmaps analysis and Pearson’s correlation analyses were performed among the studied maize hybrids (YH-5427 and FH-1046). Person’s correlation was developed (based on a two-tailed test) via R Software (version 4.3.1) to evaluate the positive and negative correlation between the attributes studied and also to assess their level of significance (*p* ≤ 0.05) with respect to each other. Pearson’s correlation performed for hybrid maize YH-5427 (Figure 8A) revealed that TFFA had a significant positive correlation with SL (r = 0.93 **), SFW (r = 0.71 **), SDW (r = 0.62 *), NaS (r = −0.9 **) and NaR (r = −0.8 **). Similarly, NR (r = 0.46) intimated a strong significant positive correlation with RL (r = 0.6 *), SFW (r = 0.97 **) (Figure 8A). Nevertheless, in the second hybrid of maize (FH-1046), SL (r = 0.99 **), SDW (r = 0.98 **) and Chl. a (r = 0.78 **) depicted a significant positive correlation with SFW (r = 0.99 **) and a negative correlation with NaS (r = −0.9 **) and NaR (r = −0.61 *). Despite this, RL (r = 0.66 *), SFW (r = 0.25) and NOL (r = 0.69 *) divulged a significant positive correlation with RDW (r = 0.39) (Figure 8B). In short, statistical analyses (PCA, Heatmap, Pearson’s correlation and ANOVA values) showed that changes in treatment level also induce alterations in morpho-physiological, biochemical and nutrient content. All these analyses showed that salinity stress (5 dS m^−1^) had drastic effects on plant vegetative growth and, ultimately, retarded plant growth by altering enzymatic activities within the cells. However, the foliar application of CuO-NPs boosts the plant metabolic process, which leads to improved growth patterns compared to plants grown under salinity stress (T_3_) and a combination of CuO-NPs + 5μM salinity stress treated maize plants (T_4_).

**Figure 9:**
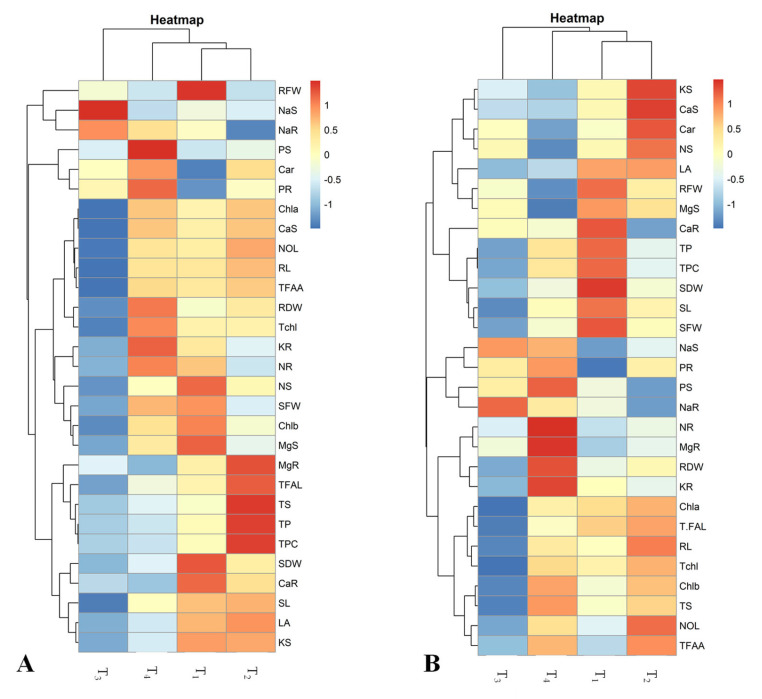
Heatmap correlation analyses of both maize hybrids (**A**) V_1_: YH-5427 and (**B**) V_2_: FH-1046 for all growth and physio-biochemical parameters where 1,2,3,4 depict T_1_, T_2_, T_3_, and T_4_ treatments, respectively.

The heatmap analysis conducted for hybrid YH-5427 showed two main clusters for applied treatments. In the first cluster, various attributes were grouped under salinity stress (T_3_) and were negatively correlated with most of the traits except sodium content in the shoot and root of the growing hybrid (Figure 9A). However, in the second main cluster, three treatments were grouped where a combination of CuO-NPs + 5 μM salinity stress (T_4_) indicated a strong positive link with shoot phosphorus (PS) and roots potassium concentration (KR), while a slight negative relationship with root magnesium ions (MgR) was manifested. In addition, the hybrid YH-5427 grown under the control condition (T_1_) showed a strong positive correlation with root fresh weight (RFW) and shoot dry weight (SDW), while it was negative with carotenoids (Car) and the concentration phosphorus in the roots (PR). Additionally, the foliar spray of CuO-NPs (T_2_) showed a positive relationship among TPC, TS and TP and a negative interaction with the Na content of the roots (NaR), (Figure 9A).

Similarly, a heatmap developed for the maize hybrid FH-1046 showed two main clusters in which all parameters were grouped under particular treatments and showed positive and negative associations among the attributes studied (Figure 9B). In the first main cluster, maize plants grown under salinity stress (T_3_) indicated a positive interaction with sodium content in roots (NaR), while a strong negative association with chlorophyll a concentration (Chl.a), root length (RL) and total sugars (Figure 9B). However, in the second main cluster, it was observed that the foliar treatment of CuO-NPs+salinity stress (T_4_) showed a slight negative interaction with LA, shoot Mg ions (MgS) and shoot nitrogen contents (NS), while CuO-NPs (T_2_) revealed a positive interaction among potassium content in the shoot (KS), calcium content in the shoot (CaS), and a strongly negative interaction with root Na concentration (NaR) and root phosphorus (PR). In addition, plants of hybrid FH-1046 grown under control conditions exhibited positive linkage with SDW, SL and SFW but a negative interaction with Na content in shoot (NaS) and root phosphorus concentration (PR) (Figure 9B).

## 3. Discussion

Salinity had a prominent effect on several physio-biochemical attributes such as biomass production, vegetative growth, membrane stability and photosynthetic activity [30]. Plants showed various responses to salinity stress; however, it has deleterious effects on whole plants at the cellular level [31]. Under stress conditions, plants try to improve tolerance by altering their physiological and biochemical processes. Under saline conditions, turgor losses have often been observed which cause lethal effects on plant growth owing to the development of hypertonic conditions around the cell. In combating salinity stress, nanoparticle application plays a pivotal role in agriculture because, through this approach, antioxidant production increases or reactive oxygen species (ROS) synthesis is retarded. The key characteristic of widely used nanoparticles is their size (ranges from 1 to 100 nm) owing to which they can easily diffuse through small membrane channels made up of proteins and lipids [32]. Several studies reported that the application of nanoparticles favored various crops, that is, the quality of citrus fruit juice [33], cucumber, tomato, mustard, Arabidopsis and maize crops to adapt under different environmental conditions [34] depending on plant species, its age and the concentration of metal nanoparticles [35].

Previous studies showed that CuO-NPs induced resistance in plants against biotic and abiotic stresses [36]. In the current investigation, the use of CuO-NPs improved the content of chlorophylls a, b and the total chlorophyll but not the carotenoid content (Figure 2) in both hybrids under saline conditions, as also reported by Shah et al. [37]. Foliar application of CuO-NPs plays a crucial role in the improvement of vegetative growth by enhancing the rate of photosynthesis and consequently improving the shoot and root lengths, and therefore, the fresh and dry biomass of the maize hybrids grown under saline conditions improved (Figure 1D,F). Salinity reduced the root length (Figure 1B), shoot length (Figure 1A), shoot dry weight (Figure 1E), shoot fresh weight (Figure 1C), number of leaves (Figure 1H) per plant (Figure 1H) and leaf area index (Figure 1G), but the foliar application of CuO-NPs improved all the parameters mentioned above in both maize hybrids, and the hybrid YH-5427 performed better than FH-1046. Previous studies also showed that growth, fresh and dry weights of the shoot and root and the number of leaves were highly sensitive to salinity stress leading to severe damage to the mesophyll region of the leaves, negatively affecting the biomass production of maize plants [38], because the reduction in the number of leaves and the leaf area significantly reduced the photosynthesis activity resulting in a reduction in growth and biomass production. All of these also altered the physio-biochemical activities at the cellular level necessary for optimal plant growth [39]. The present findings are in line with Rahman et al. [30] who observed that different responses of maize hybrids to salinity stress are due to their genetic diversity for developing tolerance to salt stress and influenced the biomass production, Na/K selectivity and gas exchange. Furthermore, metabolic changes observed under salinity stress are the most crucial factor that induces a direct impact on the productivity of the maize crop [9]. High salt stress retards physiological processes in plants, which have a pronounced influence on the reproductive stages of plant development [40]. Another investigation conducted by Vishwakarma et al. [41] showed that foliar treatment with CuO-NPs significantly improved the total protein content distributed in tomato shoots and roots.

In this investigation, salinity stress showed a greater reduction in chlorophyll a (Figure 2B) and chlorophyll b (Figure 2C) for the maize hybrid (FH-1046) compared to hybrid V_1_ (YH-5427); however, the foliar application of CuO-NPs improved all of these physiological attributes (Figure 2). There is a link between salinity-induced oxidative stress that causes antagonistic effects on plant growth, physio-biochemical, anatomical, metabolic and enzymatic activities [42]. Saline conditions alter photosynthesis due to structural variations in the chloroplast in maize crop varieties [43]. High concentrations of salt increase the chloroplasts and intercellular spaces, significantly increasing the thickness of cell walls in resistant varieties [44].

The reduction in leaf number and leaf area index alters the photosynthetic rate due to modifications in metabolic processes and chlorophyll content (Figure 2 and Figure 3). Previous studies have indicated that salinity stress reduces chlorophyll content and photosynthetic processes in plant species [45]. Similarly, maize plants maintain their physiological activities by optimizing the rate of photosynthesis and transpiration through the use of photosystems, the electron transport chain and the digestion of organic compounds through the glycolysis process [46], which fails under stress conditions and thus directly influences the growth and development of plants [47]. Similarly, total sugars (Figure 3B) and total flavonoids (Figure 3E) decreased under salinity but increased with the foliar spray of CuO-NPs for both varieties, while the same trend was observed for total protein (Figure 3C) and total phenolic content (Figure 3D) only for V_2_. Previous studies showed that the soluble sugars and protein content of shoots increases with exogenous application of CuO-NPs [48]. The latter authors found that foliar application of CuO-NPs increased all of these biochemical attributes in both of the above-mentioned hybrids, because of which maize hybrids may be able to adjust osmotically to a saline environment, and thus, foliar application of CuO-NPs was effective in minimizing the adverse effect of salinity. Previous findings have also indicated that the concentration of Cu in plants has a direct influence on the levels of soluble sugars and proteins in the shoots of studied maize plants, with a higher application of CuO-NPs leading to a significant increase in all these biochemical parameters [48].

Moreover, statistical analyses (PCA, Heatmap, Pearson’s correlation, and ANOVA values) showed that variations in all morpho-physiological and biochemical parameters studied and the content of nutrient are adversely affected by treating plants with T_3_ (salinity stress), and a decreasing trend was followed by plants that were treated with T_4_ (salinity + CuO-NPs), T_1_ (control) and T_2_ (CuO-NPs). The foliar application of CuO-NPs played a crucial role in obtaining the desired results in comparison to plants that underwent salinity stress or with a combination of CuO-NPs and salinity. The heatmap helps group the traits studied along with the specific applied treatments and represented a positive and negative correlation among several attributes (Figure 9A,B).

The reduction in morpho-physiological nutrient content and some biochemical characteristics was due to escalating levels of sodium concentration. Previous studies have demonstrated that salinity stresses severely hampered the growth and physio-biochemical processes and essential ions such as K, P, N and Mg because the higher sodium content disrupts the metabolic activities of the growing cell and slows the synthesis of several vital proteins necessary for optimal plant growth and development [49]. Due to the salinity stress, chlorophyll molecules denature reducing the photosynthetic activity by down-regulating the expression of several crucial genes linked with the synthesis of chlorophyll molecules, magnesium ions and nitrogen contents. This adverse impact on photosynthetic machinery leads to a delay in overall plant growth and activation of key enzymes to accelerate better plant growth and development [50].

The present findings demonstrated that the total protein content of the shoots (Figure 3C) improved compared to roots in plants treated with CuO-NPs, confirming what Al-Hakimi and Hamada found [51]. Previous findings indicated that plants treated with Cu did not show any significant change in protein content [52] because protein synthesis is directly controlled by certain genes. In the current study, a synergistic relationship was found between flavonoid concentration and CuO-NPs treatment (Figure 4E). The accumulation of flavonoids plays an important role in the protective responses of higher plants to various abiotic stresses, including drought, cold and high temperatures [7]. Flavonoids have been shown to have a significant influence on auxin transport within cells and in the apoplast region, which is directly related to plant height and the development of secondary growth of plants [53].

The maize hybrid YH-5427 showed resistance to salinity stress, which is why it had better plant growth compared to the hybrid FH-1046 which after receiving CuO-NPs showed comparable performance under saline conditions (Figure 1). Salinity treatment increases sodium content in the shoot of maize hybrids and decreases K, P, N, Ca and Mg content in both maize hybrids (Figure 4). Under salinity stress, the increase in Na ion concentrations is obvious, which catalyzes crystal formation inside the cell organelles or the cytoplasmic region due to the accumulation of excessive sodium ions [54]. The current study showed that under both control and salinity stress conditions, the hybrid YH-5427 had a higher content of K and N, compared to the hybrid FH-1046, which retained a higher concentration of P, Ca, Mg and Na in the roots (Figure 5). Therefore, under saline conditions and foliar application of CuO-NPs, the hybrid YH-5427 showed better adaptability and growth rate compared to the hybrid FH-1046 because elevated levels of K and N contents under salinity stress decreased Na ion uptake. Higher Na contents were observed for the hybrid FH-1046 (Figure 5A), which adversely affected its growth, development and antioxidant production to counter ROS within plant cells. Furthermore, elevated levels of calcium ions could trigger osmolytic synthesis and accumulation in plants stressed by salinity to reduce the toxicity of Na ions [55]. Exogenous application of copper oxide nanoparticles enhances nutrient uptake and other antioxidants such as total soluble proteins, total soluble sugars and proline [56].

Similarly, salinity stress improved the content of Na, P and Ca in the roots of both maize hybrids and reduced the content of K, N and Mg (Figure 5). The main reason behind salinity stress is the alteration of Na+/K+ levels because it has a direct influence on physio-biochemical processes within the plant cell. Hassanein et al. [57] observed that the accumulation of Na+ and Cl- in roots led to nutrient imbalances in root tissues and a reduction in the K+/Na+ ratio with increasing salinity stress, and similar results were found in salt-sensitive maize varieties. The salt-tolerant maize hybrid (YH-5427) maintains a high K+/Na+ in its shoots (Figure 4A and Figure 5A) and an increase in Na+ in the shoot is accompanied by a reduction in K that led to a decrease in the K+/Na+ ratio [22]. The current study revealed that, under saline conditions, the application of CuO-NPs enhances the K, N and Ca content in the hybrid YH-5427, while hybrid FH-1046 maintained a higher content of P, Mg and Na (Figure 5). In summary, under saline conditions, the hybrid YH-5427 showed better morpho-physiological and biochemical activities compared to hybrid FH-1046, which resulted in more promising results obtained for the hybrid YH-5427.

## 4. Materials and Methods

### 4.1. Experiment Layout

This investigation was carried out in a completely randomized design (CRD) at the Botanical Research Area, the University of Lahore, Lahore, Pakistan. For this study, seeds of two different maize hybrids, i.e., YH-5427 (V_1_) and FH-1046 (V_2_), were obtained from the Ayub Agriculture Research Institute, Faisalabad, Pakistan. The net-houses experiment was carried out using 24 plastic pots (pot size: diameter: 30 cm; height: 40 cm) containing 5 kg of loamy soil, whose main characteristics are summarized in Table 3. Five seeds were sown at equal distances in each pot during the second week of August 2022. The temperature range during the experimental test was 20–30 °C and the average humidity was 70%.

The treatments, replicated three times, were as follows:

(1) T1 (Control): irrigation with distilled water and no foliar application of CuO-NPs at two stages of development, i.e., six and nine leaves with visible leaf collars;
(2) T2 (Control + CuO-NPs): irrigation with distilled water and foliar application of a 5 µM suspension of CuO-NPs at two stages of development, i.e., six and nine leaves with visible leaf collars;
(3) T3 (Salinity): irrigation with a solution of NaCl with an EC value of 5 dS m^−1^ and no foliar application at two stages of development, i.e., six and nine leaves with visible leaf collars;
(4) T4 (Salinity + CuO-NPs): irrigation with a solution of NaCl with an EC value of 5 dS m^−1^ and foliar application of a 5 µM suspension of CuO-NPs at two stages of development, i.e., six and nine leaves with visible leaf collars.

All pots were fertilized according to local good agricultural practices 30 days after sowing. In detail, nitrogen was applied at a dose of 100 kg N ha^−1^ using urea and diammonium phosphate, and the latter fertilizer also provided P at a rate of 50 kg P_2_O_5_ ha^−1^, while K was applied as sulphate of potash at a rate of 50 kg K_2_O ha^−1^.

For biochemical analysis, the plants were harvested at twelve leaves with visible leaf collars stage (70 days after sowing). During the trial, the plants were regularly irrigated with tap water when necessary.

### 4.2. Growth Parameters

After harvesting, shoots and roots were separated with the help of sharp scissors. The length of the shoot and root was observed with the help of a measuring scale in centimeters. Fresh and dry weights of shoots and roots (oven-dried at 65 °C for 48 h) were determined with a digital scientific electrical balance (ATX224R, SHIMADZU, Kyoto, Japan). The flag leaf of each plant per replication was selected for leaf area measurement. The leaf area was determined by measuring leaf length (L) and leaf width (W) through scale using the formula: leaf area (cm^2^) = L × W × 0.75 [58].

### 4.3. Biochemical Parameters

The randomly selected leaves were used to determine the chlorophyll and carotenoid content extracted in an 80% acetone solution [59]. The absorbance of the extracts was measured at wavelengths of 480, 645 and 663 nm using a spectrophotometer SP-UV52N (Wincom Company Ltd., Changsha, China). The concentration of chlorophyll a (Chl a), chlorophyll b (Chl b), carotenoids (CAR) and total chlorophyll (T Chl) was estimated using the formula described by Davies [60]:

Chl a = [12.7 (OD_663_) − 2.69 (OD_645_)] × V/1000 × W

Chl b = [22.9 (OD_645_) − 4.68 (OD_663_)] × V/1000 × W

Total Chl = [20.2 (OD_645_) − 8.02 (OD_663_)] × V/1000 × W

Carotenoids = Acar/Em100%

Acar = [OD_480_ + 0.114(OD_663_) − 0.638(OD_645_)] × V/1000 × W

E100% cm = 2500

Total soluble sugars (TSS) were evaluated in leaf ethanolic extract using the protocol of Riazi et al. [61], and total soluble proteins (TSP) were estimated according to Lowry et al. [62]. Total free amino acids (TFAA) were determined according to the method of Hamilton and Van Slyke [63]. The total phenolic content (TPC) of the leaves was determined using the Folin–Ciocalteau reagent depicted by Singleton and Rossi [64]. The total flavonoid contents (TFAL) were evaluated following the Sofowara [65] and Harbrone [66] methods.

### 4.4. Nutrients

The dry material of the root and shoot was digested using the Wolf protocol [67], and the extract was used to determine the Na, K, Ca, P, Mg and N contents. The phosphorus (P) content was estimated spectrophotometrically [68], potassium (K) and sodium (Na) were assessed by the Flame photometer (Jeway, P70, Fuzhou, China) and Ca and Mg by ICP-OES (Optima 2100 DV Perkin-Elmer, Waltham, MA, USA). The nitrogen (N) content was measured according to Bremner [69].

### 4.5. Characterization of Copper Oxide Nanoparticles

**Figure 10:**
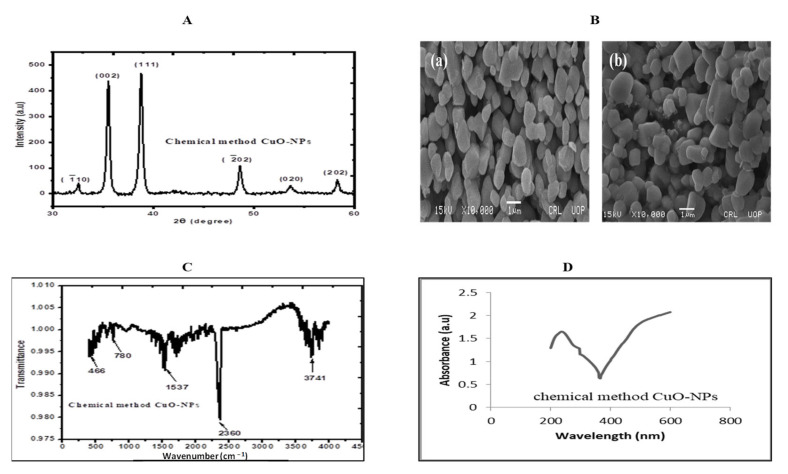
Spectra and image of copper oxide nanoparticles. (**A**) XRD analysis, (**B**) Scanning electron microscopy: indicating high resolution images of shapes of nanoparticles (a) and their spatial variations in chemical compositions (b), (**C**) FTIR and (**D**) UV visible.

The copper oxide nanoparticles were prepared by the Physics Department, GC University, Faisalabad, Pakistan [70]. To estimate the crystalline structure of the particles, CuO-NPs were subjected to XRD analysis (Figure 10A). The lattice constant and D spacing were calculated using Scherrer’s equation (d = Kλ/β cosθ), and together with data obtained from diffracted peaks, the crystal size of CuO-NPs was about 20 nm. During the next step, the surface morphology of nanoparticles was studied through scanning electron microscopy. Moreover, SEM analysis was also performed, depicting TEM images of CuO-NPs with specific micrograph sizes. Additionally, SEM images revealed a uniform distribution of the nanoparticles studied (Figure 10B), with a monoclinic shape and aggregated in the form of clusters. FTIR spectroscopy confirmed the synthesis of CuO-NPs (Figure 10C). Lastly, the absorption spectrum indicated a peak between 200 and 300 nm (Figure 10D).

### 4.6. Statistical Analysis

The data collected for all attributes studied in both maize hybrids were analyzed using an experimental CRD design for analysis of variance (ANOVA) to assess their significance. The means were compared using Tukey’s test at a probability level of 5% using the XL-STAT software (version 2019). Pearson’s correlation, Principal component analysis (PCA) and heatmaps were developed separately for both maize hybrids by using R software (version 4.3.2).

## 5. Conclusions

The effect of salinity is not good for plant growth, and this has also been demonstrated in the present work. In fact, our results indicated that salinity adversely affected a lot of physiological parameters, such as total free amino acids, total phenolic content, total proteins, photosynthetic activity and total chlorophyll content. The foliar application of copper oxide nanoparticles helped to overcome the salinity stress enhancing the aforementioned parameters in both hybrids of maize. However, the behavior of the two hybrids differed when under salinity stress, with the YH-5427 hybrid more resistant to such stress than the FH-1046. This could be possibly related to a better ratio between Na and other nutrients, such as K, resulting from the foliar application of CuO-NPs.

In the present study, we sprayed nanoparticles at six and nine leaves with visible leaf collars, and the results were quite encouraging. Therefore, it might be possible to counteract the negative effect of salinity by foliar application of CuO-NPs at specific phenological stages of the crop. However, further investigations are needed regarding the application at other phenological stages and the concentration of nanoparticle suspensions.

## Figures and Tables

**Table 3:** Characteristics of soil used in this experiment.

Soil Characteristics	Values
Soil Texture	Loamy
EC (dS m^−1^)	0.5
pH	7.8
Organic matter (%)	0.4
Available Phosphorus (ppm)	2.4
Extractable Potassium (ppm)	45.0

## Data Availability

Data are contained within the article.
